# Unlocking Innovation: How absorptive capability unleashes the potential of motivation and leadership to catalyze innovative work behaviors in Batam

**DOI:** 10.12688/f1000research.148807.1

**Published:** 2024-06-19

**Authors:** Eduard Sinaga, Suparto Wijoyo, Yetty Dwi Lestari, Fendy Suhariadi, Adrid Indaryanto, Dwi Bambang, Muhaimin Hikmah

**Affiliations:** 1 Doctoral Student Program of Human Resource Development, Postgraduate School, Universitas Airlangga, Surabaya, East Java, Indonesia; 2 Vice Director III Postgraduate School, Professor in Faculty of Law, Universitas Airlangga, Surabaya, East Java, Indonesia; 3 Doctoral Program of Human Resource Development, Postgraduate School, Associate Professor of Faculty of Economics and Business, Universitas Airlangga, Surabaya, East Java, Indonesia; 4 Head of Doctoral Program of Human Resource Development, Postgraduate School, Professor in Faculty of Psychology, Universitas Airlangga, Surabaya, East Java, Indonesia

**Keywords:** Absorptive capability, Job motivational, Transformational Leadership, Innovation work behavioral, freelancer

## Abstract

**Background:**

The significance of innovative work behavior is growing as organizations strive to maintain their competitiveness by hiring freelancer employees. This study aimed to examine how absorptive capability mediates the connections between job motivation, transformational leadership, and innovation work behavior in freelancers working in the oil and gas industry.

**Methods:**

A quantitative methodology was employed through an online questionnaire, using a random sampling strategy involving 250 respondents. Furthermore, data analysis was performed using structural equation modelling.

**Results:**

The findings indicate that job motivation and transformational leadership did not have a direct impact on innovation work behavior. However, they did have an indirect beneficial influence mediated by absorptive capability. This study revealed that absorptive skills play a vital role in facilitating the translation of job drive and transformational leadership into innovative work behaviors.

**Conclusions:**

The practical consequences involve firms embracing transformational leadership and establishing training programs to enhance employees' absorptive potential, thereby promoting innovation. Ultimately, cultivating absorptive skills is crucial for leadership styles and motivational elements to be evident in innovative work behaviors.

## Introduction

Employee innovation work behavior has become a very important foundation for supporting the success of a company. An employee’s Innovation work behavioural is defined as a behavior to create, introduce, and implement ideas deliberately within the scope of a work role, group, or organization ( Janssen, 1998; Scott & Bruce, 1994).

To achieve a high level of innovation, organizations need to improve their employees’ competitiveness by enhancing their knowledge, skills, and ability to perform multiple and diverse tasks ( Dong et al., 2017) within the scope of an organization. Competition among companies in various sectors drives many companies to improve their efficiency, flexibility, agility, and innovation in employment ( Michie & Sheehan, 2003). Competition between companies in various sectors encourages many companies to improve efficiency, including employee involvement in innovation work behavior, especially in the oil and gas industry.

Previous research has widely used the concept of absorptive capability in analyses at the organizational level ( Cohen and Levinthal, 1990; Kamal and Flanagan, 2014). Innovation work is a deliberate proposal and application of new and better ideas, processes, practices, and policies aimed at organizational efficiency and long-term sustainable business success ( Anderson et al., 2014).

Shafi et al. (2020) examined how transformational leadership is related to employee Innovation work behavioural through intrinsic job motivational, psychological empowerment, and creative process involvement and in addition The research results of Shin, et al. (2017) proved that job motivation has a positive effect on employee performance, whereas job satisfaction is independent.

In the oil and gas industry, several journals discuss absorptive capability as a supporter of innovation work behavior in the oil and gas industry ( Ahmad, & Azmi, 2016), but not supported by job motivational and transformative leadership. Absorptive capability interacts with work environment factors to influence innovative work behavior of employees in the Vietnamese oil industry ( Nguyen & Simkin, 2020), and the oil and gas company in the Middle East shows a positive influence of absorptive capability on the company's ability to innovate ( Al-Husseini, & Elbeltagi, 2018), with the objective of permanent employee research that the role of freelancers has not been found in this study.

The oil and gas industry has permanent workers hired based on long-term strategies ( Esswein et al., 2014). Have a multi-year or permanent working bond to ensure continuity of knowledge and experience in supporting long-term productive assets, and provide technical and managerial training to improve competence in line with technological developments and challenges of the oil and gas industry entitled to health benefits, bonuses, insurance, pension plans, and other facilities ( Ramanigopal, 2013).

However, at present, many oil and gas work projects are short-term, so organizations must innovate with innovative solutions using freelancer labor with consideration of cost efficiency, fresh and more objective ideas, skilled personnel, and always updated, flexible, and good service ( Shkurupii et al., 2021).

Freelancers are defined as unemployed or end-dependent contractors or may be referred to as private entrepreneurs because they do not have a permanent employment contract with whom they are employed and usually do not receive a fixed salary but are paid at a certain cost for the work they do ( Burke & Cowling, 2020).

By providing new insights to employees, thus leading to innovation work behavior ( Ávila, 2022), it is imperative to link this research with the employee status called Freelancer. Based on the explanation above, there are three potential variables that need more investigation to achieve and measure innovation work behavior: absorptive capability, job motivation, and transformative leadership. However, the Freelancer in the O&G industry context has become crucial for further discussion because of the urgent need to support the company's competitive advantage and the number of freelancers who are increasingly more numerous than permanent employees.

Furthermore, this research is expected to be academically able to fill the research gap on the influence of job motivational and transformational leadership on innovation work behavioral freelancers with absorptive capability as a variable mediator in oil and gas mines, and is expected to provide a solution contribution to the organization of companies working on projects with the needs of human resources at the international level.

### Literature

Innovation work behavioural refers to the intentional introduction and application of new ideas, processes, products, or procedures within one's work role, work unit, or organization ( Janssen, 2000; Messmann & Mulder, 2011). As workplaces face increasing complexity and competitiveness, innovative work behavior is essential for organizational success and survival ( Anderson et al., 2014). Employees must continuously seek and implement new approaches, knowledge, and technologies to improve their work performance, effectiveness, and adaptive capacities.

Based on the previous literature review and model development, seven hypotheses were proposed to test the direct and indirect effects of job motivational and transformational leadership on absorptive capability and innovation work behavior to answer the question of whether the independent variables have a significant effect either independently or through the mediating role of absorptive capability in translating their influence on employee innovation work behavior.

### Hypotheses development

Extrinsic and intrinsic motivations are fundamental factors in the theory of motivation ( Chang et al., 2021; Ryan & Deci, 2000). Extinctic motivation refers to motivation driven by external factors, whereas instinctive motivation relates to personal motivation that originates from within oneself ( Ryan & Deci, 2000). The motivating factor for a person to choose to be a freelancer is that they have flexible work activities that can be done anywhere ( Riyono & Usman, 2022). Absorptive capability has been marked as one of the most important abilities of multinational corporations, and in analyzing the ability of individuals to recognize, assimilate, and exploit new knowledge of forms of job motivation, namely instrumental and extrinsic, found significant positive relationships between absorptive ability and job motivation ( Yildiz et al., 2019; Murovec & Prodan, 2009; Sancho-Zamora et al., 2022). An empirical study by Yildiz et al. (2019) suggest that the nature of innovation work behavior is complex and not fundamental enough to produce such behavior with only instrumental and extrinsic motivation. Individuals who are intrinsically or extrinsically motivated tend to have a natural interest in learning new things, exploring innovative ideas, and implementing the knowledge acquired to improve their performance. Hypothesis 1 can be submitted as follows:

H1: Job motivational has a significant positive effect on absorptive capability.

Organizational leadership styles diversify internal corporate knowledge and enable organizations to acquire and assimilate new external knowledge to enhance corporate creativity and innovation ( Mokhber, Khairuzzaman and Vakilbashi, 2018). Absorptive capability allows companies to increase their speed, frequency, and level of innovation, which means that leadership is an ideal factor for companies to categorize, understand, and connect external knowledge to create more qualitative innovation ( Leal-Rod, et al., 2014). Transformational leadership, characterized by inspiring and motivating qualities for followers, can boost the ability of organizations to absorb, assimilate, and apply new knowledge ( Setyadi et al., 2023). This ultimately leads to an increase in absorptive capability; thus, Hypothesis 2 can be put forward as follows:

H2: Transformational leadership has a significant positive effect on absorbing capacity.

This study is also supported by research on the relationship between employee motivation towards innovation work behavior of R&D employees in three high-tech organizations with the results of the analysis of job motivational positive influence on employee innovation ( Saether, 2019). Job motivation influences employee Innovation work behavioural is as follows: the higher the employee's motivation, both intrinsic and extrinsic, the higher the tendency to engage in innovative conduct such as producing ideas, products/processes, or new solutions that are beneficial to the Enkel et al. (2017), Kasımoğlu and Ammari (2020), and Kwon and Kim (2020).

H3: Job motivational has a significant positive influence on Innovation work behavioural

The results of Lin's study (2023) show that (1) transformational leadership has a positive impact on innovative work behavior, (2) the identification of organizations/employee voices mediates the relationship between transformative leadership and innovative working behaviors, and (3) the indirect influence of transformational leadership on innovation work behavior (through organizational/employee voice identification) is stronger when employees work in more innovative climates. This study provides hotel management insights into why and under what conditions employees behave in this manner. Thus, Hypothesis 4 can be submitted as follows:

H4: Transformational leadership has a significant positive influence on employee Innovation work behavioural

Absorptive capability is defined as a set of organizations that routinely and in a company's process, acquire, assimilate, transform, and exploit knowledge to produce organizations with dynamic capabilities ( Zahra and George, 2002). Absorbency is the ability of an organization to exploit external knowledge ( Grünfeld, 2003), initiate change from within, and identify and assist ideas from the external environment ( Lewin, et al., 2011) as a dynamic ability that enables an organization to build competence ( Zahra dan George, 2002). The higher the capacity of employees and organizations in absorbing new external knowledge and applying it, the higher the employee's innovative behaviour, such as generating new useful ideas/products/processes Sethibe and Steyn (2017) and Surucu et al. (2021), can be proposed as follows:

H5: Absorptive capability has a significant positive influence on employee Innovation work behavioural.

Absorptive capability is defined as a set of organizations that routinely and in a company's process, acquire, assimilate, transform, and exploit knowledge to produce organizations with dynamic capabilities ( Zahra and George, 2002). Absorbency is the ability of an organization to exploit external knowledge ( Grünfeld, 2003), initiate change from within, and identify and assist ideas from the external environment ( Lewin, et al., 2011) as a dynamic ability that enables an organization to build competence ( Zahra dan George, 2002). Absorptive capability capable of mediating the influence of Job motivational is the motivation of work that drives the improvement of the absorptive capability of individuals and organizations. Increased absorptive capability facilitates the application of new knowledge in the form of innovation work behavior by employees, and Hypothesis 6 can be submitted as follows:

H6: Absorptive capability positively mediates the relationship between job motivation and IWB.

Absorptive capability is defined as a set of organizations that routinely and in a company's process, acquire, assimilate, transform, and exploit knowledge to produce organizations with dynamic capabilities ( Zahra and George, 2002). Absorbency is the ability of an organization to exploit external knowledge ( Grünfeld, 2003), initiate change from within, and identify and assist ideas from the external environment ( Lewin, et al., 2011) as a dynamic ability that enables an organization to build competence ( Zahra dan George, 2002). Absorptive capability is capable of mediating the influence of transformational leadership on Innovation work behavioural. Transformative leadership increases the absorptive capabilities of organizations and employees. Increased absorption capacity further facilitates the application of new knowledge in the form of employee innovation behavior.

H7: Absorptive capability positively mediates the relationship between transformational leadership and employee Innovation work behavioural.

## Methods

### Study design and participants

In order to better understand the connections between innovative work behavior, transformational leadership, absorptive capacity, and job motivation among independent contractors in the oil and gas sector, this study uses a quantitative research methodology. Using an online survey, information was gathered from a sample of 250 independent contractors operating in Batam, Indonesia. The study variables were measured using validated scales in the questionnaire, which had a 5-point Likert-type response format. The period of data gathering in 2023 was June through October.

The questionnaire was tested with a small sample of independent contractors in order to account for any biases and guarantee the accuracy of the results. It was then modified in response to their input. A randomly chosen sample received the online questionnaire via email, and participants were guaranteed anonymity and confidentiality. We acquired informed consent from each individual.

Using AMOS 26.0 software, structural equation modeling (SEM) was used to analyze the gathered data. Several relationships between variables can be examined simultaneously while accounting for measurement error with SEM. The study used a two-step methodology: first, confirmatory factor analysis was used to assess the measurement model, and then, the structural model was examined to test the proposed links.

The population in this study was freelancer employees or freelancers working in the oil and gas industry under PT. NES Indonesia, PT. Brunel Indonesia and Spencer Ogan as many as 500 Freelancer employees with a wide range of educational backgrounds, qualification and competences who being working at several oil and gas worldwide company, join a wide variety of positions, And obtained as many as 250 valid respondents, data collection starts from June 2023 until October 2023.

### Data collection

Data collection techniques are the methods used by researchers to collect data from their researchers. As for data collection techniques, a questionnaire is a data collection technique that is carried out by giving a set of questions or written statements to respondents to be answered online via email. Research data is also obtained from reports and other documents, which is closely related to the object of research and reading literature as the theoretical basis that will be used as a theoretical foundation in this research for a four-month period.

A 13-item scale that was modified from Aga et al. (2016) and Ferreras-Méndez et al. (2018) was used to measure transformational leadership. Four aspects of transformational leadership are evaluated by the scale: intellectual stimulation, customized consideration, inspirational motivation, and idealized influence. “My supervisor seeks differing perspectives when solving problems” (intellectual stimulation) and “My supervisor communicates a clear and positive vision of the future” (inspirational motivation) are two examples of example items. On a five-point Likert-type scale, participants indicated how much they agreed with each statement.

A 10-item scale that was modified from Ávila (2022) and Duan, Wang, and Zhou (2020) was used to test absorbtive capability. The scale evaluates the capacity of an organization to obtain, incorporate, modify, and apply new information. Examples of such statements are “Our organization can apply new knowledge with the required skills” (transformation) and “Our organization can work more efficiently by implementing new knowledge” (exploitation). On a five-point Likert-type scale, participants indicated how much they agreed with each statement.

A nine-item scale that was modified from De Jong and Den Hartog (2010) and Bourini (2021) was used to measure innovative work behavior. The measure evaluates a person's capacity to create, advance, and implement innovative ideas at work. “I generate original solutions for problems” (idea creation) and “I transform innovative ideas into useful applications” (idea realization) are two examples of example items. On a five-point Likert-type scale, participants indicated how much they agreed with each statement.

The complete list of questionnaire items used in this study is accessible at https://doi.org/10.6084/m9.figshare.25271419.v1 in the supplemental materials. All of the scales were chosen based on their proven psychometric qualities and prior application in comparable research settings, in order to reduce any potential biases and guarantee the validity and reliability of the measurements. Furthermore, before to being distributed to the entire sample, the questionnaire was tested on a limited group of independent contractors to evaluate its comprehensiveness and lucidity.

### Measure for validity and reliability

The degree to which the indicators employed to gauge a certain latent concept are actually related to one another is known as convergent validity ( Franke et al., 2021). An analysis of the factor loadings was conducted to assess convergent validity. According to Lee (2019), loading factors are coefficients that assess how latent variables, also known as constructs, relate to their indicators. The indicator measures the construct, as shown by a strong factor loading. Loading factors are used in the validity and reliability tests of this study; an outer loading value of more than 0.5 indicates validity ( Rodriguez et al., 2016).

Confirmatory factor analysis was used in this study to assess the measurement instrument's concept validity. The analysis's findings demonstrate that each construct variable indicator had significant loading factors that were all more than 0.5. These findings offer compelling proof of the study instrument's great construct validity. According to the study's theoretical framework, the instrument's high construct validity score suggests that it is successful in measuring the desired variables.

Construct Reliability (CR) values larger than 0.7, on the other hand, suggest that an instrument has a high degree of consistency when assessing the intended construct in the context of reliability testing ( Marsh et al., 2014). When testing the same construct again on the same sample, the instrument or questions in the instrument are consistent if the Construct Reliability (CR) value is higher than 0.7. Stated differently, the tool produced consistent and dependable results.

The Average Variance Extracted (AVE) value of each variable in this study was used to determine the Construct Reliability (CR) values, which were found to be greater than 0.7 based on the results of CR analysis. These results show that the data are trustworthy and that the Construct Reliability criteria is satisfied. If the measuring tool were to be used repeatedly on the same sample, the high CR values indicate that it has a high degree of internal consistency and would yield results that are comparable.

For the study's conclusions to be accurate and reliable, the measurement model's convergent validity and reliability are essential. The significant factor loadings show how well the indicators capture the desired variables and how closely they connect to the corresponding constructs. The measuring scales' internal consistency and reliability are further supported by the high CR values, which instill trust in the data's outcomes and conclusions.

### Model fit testing

The results of the comprehensive model fit test showed that the model used was very good at explaining the existing data. To measure the degree of model fit, several key parameters were used, all of which showed good results. First, the Chi-Square/DF, which is one of the main indicators for measuring model suitability, has a value of 2.326. This value is much smaller than five, which indicates that the model has a very good degree of fit. In addition, the chi-square p-value is 0.000, which is obviously smaller than 0.05; therefore, this model is statistically significant.

The RMSEA (Root Mean Square Error of Approximation) value was 0.073. The range of RMSEA values (0.07 to 0.085) also showed that this model had a very good level of fit. The RMSEA p-value is 0.000, indicating a significant level of fit. The Goodness of Fit (GoF) index also supports these results, with measures such as ECVI, AIC, and CAIC showing low values and close to a perfect model.

In addition, various model assessment indices, such as NFI, RFI, IFI, TLI, CFI, RMR, GFI, AGFI, and PGFI, all show a good level of fit because they are close to the threshold, so they can be in the moderate and close fit categories. Based on these positive results, it can be concluded that this model is very good at explaining the data used in this study. The strong level of fit between the model and data confirms that this model can describe the relationships between the variables studied very well.

### Structural model analysis

The data analysis in this study using software program AMOS version 26 for the Structural Equation Modelling. The alternative free software of the Structural Equation Modelling would be the package “lavaan” in R, which is available for free on the open-source statistical computation environment R accessible at https://www.r-project.org/.

### Ethical considerations

This study was conducted in accordance with the Declaration of Helsinki, which outlines the ethical principles for medical research involving human subjects. Ethical approval for this study was obtained from the Universitas Airlangga Research Ethics Committee on (protocol number: 779/UN3. SPS/I/PT.01.06/2023 All participants were informed about the purpose and procedures of the study, and their rights as participants. Written informed consent was obtained from all participants prior to their involvement in the study. Participants were assured that their participation was voluntary, and they could withdraw from the study at any time without consequence. All data collected during the study were kept confidential and anonymized to protect participants' privacy."

## Results

The results of the tests revealed the direct and indirect effects of the independent variables on the dependent variable. In particular, job motivational and transformational leadership as independent variables are thought to affect absorptive capability and innovative work behavior as dependent variables, either directly or indirectly, through mediating relationships. The test results are described in more detail in the following paragraphs to provide a comprehensive picture of the pattern of relationships between variables in the proposed research model. Thus, the proof of the previously formulated hypotheses can be presented systematically to show the validity of the model and provide theoretical and practical implications for the research results.

Hypothesis testing aims to determine the relationship between independent and dependent variables. There is a significant relationship if the significance value is below 0.05, the critical ratio (CR) value is above the table (1.96). The hypothesis is tested by analyzing the estimated number of influences between variables in the model, and the estimated value was considered significant if the significance level (sig p) was ≤ 5%.

### Participants

The majority of respondents were men (90.8%) and only 9.2% were women. Regarding educational level, the majority had S1 education (57.2%), followed by SLTA (21.6%), D3 (14.4%), and S2 (6.8%). The respondents were mostly over 35 years old (74.8%), 21.6% were 25-35 years old, and only 3.6% were under 25 years old. In terms of work experience, 29.2% had worked for 6-10 years, 27.6% for 11-15 years, 20.4% over 21 years, 15.2% from 1 to 5 years, and 7.6% had been working for 16-20 years, leading to the list characteristics in the link https://doi.org/10.6084/m9.figshare.25271281.v1.

### Direct effect

Here are the results of the direct influence test presented in Table 1:

**Table 1.** Direct effect result.

Direct effect	Estimate	SE	CR	P-Value
Absorptive Capability <--- Job motivational	0.715	0.178	4.015	0.715
Absorptive Capability <--- Transformational Leadership	0.392	0.104	3.749	0.392
Innovation Work Behavioural <--- Absorptive Capability	0.686	0.118	5.816	0.686
Innovation Work Behavioural <--- Job motivational	0.049	0.142	0.344	0.495
Innovation Work Behavioural <--- Transformational Leadership	0.03	0.092	0.328	0.307

The results of the direct effect analysis show the direct influence of independent variables (job motivational and transformative leadership) on the dependent variable (absorptive capability and innovation work behavior), as described below:

Absorptive capability <--- Job motivation (0.715, p < 0.001): Job motivation has a significant positive influence on absorptive capability (H1 Received). With a coefficient of 0.715, any increase in job motivation by one unit will result in an increase of approximately 0.715 units in absorbing capacity. Absorbing Capacities < Transformative Leadership (0.392, p < 0,001): A significant positive impact of transformative leadership on absorbing capabilities (H2 Receiving). With an increase in job motivation by 0.392, every increase in transformational leadership by one unit will lead to an increase of approximately 0.392 units of absorptive capability. Innovation work behavioral--- < 0.686, p< 0.001: A significant negative impact of the significant absorbant capacity on inactivity will not be considered to be greater than the value of inefficiency (H3Received in relation to work).

Innovation work behavioral <--- Transformative Leadership (0.03, p > 0.05): The influence of transformative leadership on innovation work behavior is also not significant (H5 Rejected). P-values greater than 0.05 indicate that this relationship is not significant.

### Indirect effect

Here are the results of the indirect influence test shown in Table 2:

**Table 2.** Indirect effect result.

Indirect effect	Standart error	P-Value estimate	
		Upper	Lower
Transformational Leadership ---> Absorptive Capability ---> Innovation Work Behavioural	0.138	0.083	0.547
Job motivational ---> Absorptive Capability ---> Innovation Work Behavioural	0.237	0.252	0.944

The results of the indirect effect analysis show indirect influence (through mediator) of the independent variables (transformative leadership and job motivation) on the dependent variable (innovation work behavior) through the mediator (absorptive capability) as follows: transformative leadage ---> absorptive capability ---> innovation work behavior (0.547, p < 0.001), and transformational leadership has a significant indirect impact on innovative behaviors through absorptive capability mediators (H6 Accepted). A coefficient value of 0.547 indicates that any unit increase in transformative leadership will result in an increase in innovation work behavior of approximately 0.547 units through intermediate absorbant capacity.

Job motivational→ absorptive capability → innovation work behavior (0.944, p < 0.001): There is a significant indirect influence of job motivation on innovation work behavior through the Absorbing Capabilities Mediator (H7 Accepted). A coefficient value of 0.944 indicates that every one-unit increase in job motivation results in an increase in Innovative Conduct of approximately 0.944 units through the absorptive capability mediator.

The results of the R-squared test are presented in Table 3.

**Table 3.** R-Square result.

R	Estimate	Lower	Upper	P
Absorptive Capability	0.550	0.374	0.671	0.010
Innovation Work Behavioural	0.549	0.368	0.742	0.010

The results of this R-Square analysis provide a deeper understanding of how big the impact of absorptive capability is on innovation work behavior, and vice versa. Although the analytical model explains most of the variability in both, approximately 45% of the variation in innovation work behavior cannot be explained by the factors in the model. These factors may include other variables not included in this analysis or external factors that directly affect innovation work behavior. Therefore, this R-Square analysis validates the importance of absorptive capability and innovation work behavioral variables in the context of this research, but also shows that there are other factors that affect innovation work behavior that need to be considered.

## Discussion

The test results indicated a notable and favorable influence of job motivation on absorptive capability, as evidenced by a coefficient value of 0.715. These findings suggest that for every one-unit increase in job motivation, there is a corresponding increase of approximately 0.715 units in absorption capacity. The results emphasize the significance of job motivation as a critical element that improves the capacity of individuals and organizations to assimilate and apply new knowledge, which is essential for growth and achievement in the workplace. This study corroborates previous discoveries made by Yildiz et al. (2019), who also observed a favorable correlation between job motivation and absorptive capability in the textile industry. Additionally, this aligns with the research conducted by Ali and Park (2016), who demonstrated the beneficial influence of self-efficacy on knowledge acquisition.

Furthermore, this study elucidates the distinction between intrinsic and extrinsic motivation, as delineated by Chang et al. (2021) and Ryan and Deci (2000). Extrinsic motivation is influenced by external stimuli, whereas intrinsic motivation originates from an individual's own drive. Riyono and Usman (2022) conducted a study which found that motivation, encompassing both inner and extrinsic factors, significantly influenced the decision to pursue freelancing, with the need for job flexibility being a primary driving force. This demonstrates that desire impacts not only absorptive capability but also individual decisions regarding career paths.

As described by Zahra and George (2002), absorptive capability refers to an organization's capability to effectively obtain, integrate, convert, and leverage external knowledge. This study shows the significant impact of absorptive capability on improving organizational innovation and adaptation. This finding supports the hypothesis that absorptive capability is a crucial skill for both multinational corporations and individuals to identify and leverage new knowledge.

The test demonstrated that transformative leadership had a significant and favorable impact on absorptive capability, as evidenced by a coefficient value of 0.392 and a p-value below 0.001. This demonstrates that leadership styles that inspire and motivate can improve capacity to assimilate and exploit new knowledge. This research aligns with the findings of Mokhber, Khairuzzaman, and Vakilbashi (2018), who demonstrated that different leadership styles can enhance the variety of information within an organization and promote the integration of new knowledge from external sources.

Nevertheless, the test results indicated that job motivation did not have a substantial impact on employee innovation work behavior/, since the p-value was above 0.05. This implies that job motivation alone may not be adequate for stimulating inventive behavior, necessitating the inclusion of other elements or additional variables for a more comprehensive explanation. This discovery contradicts the research conducted by Dai et al. (2022), who concluded that individual motivation and a climate that fosters innovation have a substantial impact on students' inventive behavior.

Conversely, research has demonstrated that the ability to absorb new information and ideas has a strong and beneficial impact on one's tendency to engage in Innovation work behavioural. This relationship was quantified using a coefficient of 0.686. This statement asserts that the capacity to absorb and apply new information significantly impacts the innovative conduct of individuals and organizations. The findings of Kang and Lee (2017) and Saether (2019) further corroborate these results, demonstrating the favorable influence of absorptive capability on innovation behavior across different industries.

In addition, the test findings indicate that absorptive capability has the ability to moderate the impact of job incentives on employee inventive behavior, with a coefficient value of 0.944. This implies that, while job motivation does not directly impact inventive behavior in a substantial way, it can enhance innovative conduct by increasing absorption capacity. According to a study conducted by Cui, Wu, and Tong (2018), absorptive capability plays a role in promoting open innovation by connecting different forms of management relationships to invention.

Additionally, this study indicates that transformative leadership exerts a substantial indirect impact on innovation work behavior through absorptive capability. This finding aligns with previous research conducted by Flatten et al. (2015), Afsar et al. (2014) and Noruzy et al. (2013), who demonstrated that absorptive capability mediates between transformational leadership and innovation work behavior.

## Conclusions

Studies emphasize that absorptive capability, which refers to an individual's aptitude to identify, incorporate, and leverage new knowledge, significantly influences the motivation of freelancers to pursue and excelle in their employment. Companies can ensure their ability to adapt to changing circumstances and further enhance their knowledge and competence by employing individuals with a high level of absorption capability.

Furthermore, studies emphasize that transformational leadership significantly influences freelancers’ absorptive capability by providing a solid basis for them to explore tactics, such as training and development, that can enhance their ability to absorb new knowledge and ideas. This, in turn, leads to an increased motivation for work. This leadership style has the potential to improve freelancers' capacity to effectively identify, absorb, and integrate external knowledge, thereby elevating organizations' innovation levels.

Users who work as freelancers: A strong work ethic fosters a drive to actively pursue, comprehend, and utilize novel information, hence enhancing individuals' capacity to assimilate and effectively apply new knowledge. This, in turn, significantly influences proficiency levels. Freelancers should prioritize the enhancement of their absorptive capabilities. This can be achieved by consistently pursuing and incorporating new knowledge pertinent to their jobs. Engaging in training and self-development can enhance proficiency in several areas. Enhances Job motivation as an independent contractor.

### Limitations and future research

Research examining the correlations between job motivation, transformative leadership, absorptive capability, and innovation work behavior has yielded significant findings. These findings emphasize the positive impact of job motivation and inspiring leadership on the capacity of organizations and individuals to assimilate and utilize new knowledge, ultimately fostering Innovation work behavioural. Nevertheless, the research is subject to some limitations, including restricted generalizability of the findings, potential bias in the data provided by individuals themselves, and the use of cross-sectional designs that cannot definitively establish causality. These limitations indicate that although the findings offer useful insights into the elements that influence absorptive capability and innovation work behavior, there is still a need for additional studies to enhance our understanding of these dynamics in many situations through the use of more varied approaches.

### Future research

Future research should focus on conducting longitudinal studies to further investigate the relationship between work motivation, transformative leadership, absorptive capability, and innovation work behavior. These studies would examine the lasting impact of transformational leadership on absorptive capability and innovation in different organizational settings while also addressing the limitations of previous research. This methodology will allow researchers to evaluate the cause-and-effect relationship and patterns of change over a period of time, offering a more comprehensive understanding of how leadership interventions can effectively improve the long-term innovative capabilities of companies. Furthermore, the integration of variables such as organizational culture and individual psychological aspects might provide a fresh outlook on how internal and external factors influence or mediate these interactions. This research can assist firms in creating work environments that are more favorable to innovation by considering the aspects that impact motivation and ability to create.

Moreover, employing a mixed-method approach to comprehend the impact of digital technology on absorptive ability and innovation work behavior can provide valuable insights into the current digital era. This entails examining how transformative leadership can be adjusted and implemented in hybrid and remote work settings to uphold or amplify motivation and innovation. This research aims to address the existing gaps in the literature and offer practical counsel to managers and leaders in effectively navigating the challenges and opportunities presented by technology and evolving work environments. This study aims to enhance our comprehension and practical knowledge of strategies that effectively promote and cultivate Innovation work behavioural within firms, regardless of their sector or working conditions.

#### Ethics and consent

Universitas Airlangga Research Ethics Committee approved this study on April 17, 2023 (approval number: 779/UN3.SPS/I/PT.01.06/2023). The committee reviewed the study protocol to make sure it followed ethical principles for research involving human participants. All participants provided written informed consent prior to participating in the study.

#### Consent to participate

All freelancers who participated in the study provided informed consent by filling out the consent form in the attached questionnaire in Google Forms. Selecting the consent option provided detailed information about the purpose of the study, which was to examine the relationship between work motivation, transformational leadership, absorptive capacity, and innovative work behavior among freelancers in the oil and gas industry. The participants were informed of the necessary procedures, including completing an online questionnaire for approximately fifteen to twenty minutes, and that they could leave the study at any time without any consequences. The consent form also emphasized the voluntary nature of participation, ensuring that their responses would be kept confidential and that their data would be anonymized to protect their privacy.

The Research Ethics Committee of Universitas Airlangga has approved the written consent process as part of the research protocol (approval number: 779/UN3.SPS/I/PT.01.06/2023). The ethics committee examined the research forms and procedures to ensure that they followed the ethical principles for research involving human participants.

## Data Availability

Direct links to the particular datasets supporting your research on the connections between innovative work behavior, transformational leadership, absorptive capacity, and job motivation among independent contractors in the oil and gas sector are provided in this section. The findings of the questionnaire, the list of questions and their descriptions, and the profiles of the 250 freelancers who took part in the study are all included in the datasets, which are stored in the Figshare repository. The data underlying this study are available in the Figshare repository:
• Dataset of questionnaire results from the respondents of absorptive capability of freelancers: https://doi.org/10.6084/m9.figshare.25284934.v1 1. Figshare: List of questions and descriptions of the questionnaire - Absorptive capability mediates the connections between job motivational, transformational leadership, and Innovation work behavioural in freelancers working in the oil and gas industry: https://doi.org/10.6084/m9.figshare.25271419.v1
2. Figshare: Profile of 250 freelancers working in the oil and gas industry in Batam, Indonesia: https://doi.org/10.6084/m9.figshare.25271281.v1 Data are available under the terms of the Creative Commons Attribution 4.0 International license (CC-BY 4.0).
