# Supplementary material for: Unlocking Innovation: How absorptive capability unleashes the potential of motivation and leadership to catalyze innovative work behaviors in Batam
Source: F1000Res. 2024 Jun 19;13:663. [Version 1] doi: 10.12688/f1000research.148807.1 (PMC11480712; doi:10.12688/f1000research.148807.1)
Supplement: Peer review history

# Reviewer report: Reviewer response for version 1

**DOI:** 10.5256/f1000research.163172.r311219

**Published:** 2024-10-15

**Authors:** Yandra Rivaldo, Septa Diana Nabella

**Affiliations:** 1 Faculty of Economics and Business, Universitas Ibnu Sina, Batam, Riau Islands, Indonesia; 2 Universitas Ibnu Sina, Faculty of Economics and Business, Batam, Riau Islands, Indonesia

Dear Author.

 I have read this paper with various academic perspectives. In general, this paper is interesting, but there needs to be improvement in several aspects and improvements to the main points of the research.

 1. Introductory part - the aim of this research is stated to be to fill gaps, whereas no gaps have been stated yet. The gap does not only lie in previous results, but the gap also comes from scientific arguments in previous research or theoretical gaps between current research and previous theoretical approaches. Then, the paper should be equipped with outline data regarding field facts.

 2. The paper needs to explain the main theoretical approaches that are relevant as a basis for hypothesis development.

 3. Method - paragraph 1 250 Independent contractor, paragraph 4 250 sample, looks ambiguous, needs clarity. Then the questionnaire is distributed via email or online, the author must explain the evidence that the questionnaire was answered truthfully and to the right person, because in some cases irregularities in filling out often occur. Next, in the method section, the author needs to write the structural equation.

 4. Discussion section - there are still many repeating sentences and results contained in the results section and explaining general things. In this section the author must further explore scientific arguments based on the findings in the results section, especially the empirical impacts and implications. Next, the sentence ''absorptive capability has the ability to moderate the impact of job incentives on employee inventive behavior'' moderation or mediation?

 Thank you.

Is the work clearly and accurately presented and does it cite the current literature?

Yes

If applicable, is the statistical analysis and its interpretation appropriate?

Yes

Are all the source data underlying the results available to ensure full reproducibility?

Yes

Is the study design appropriate and is the work technically sound?

Yes

Are the conclusions drawn adequately supported by the results?

Yes

Are sufficient details of methods and analysis provided to allow replication by others?

Yes

Reviewer Expertise:

Organizational, Leadership, Behaviour, Performance

We confirm that we have read this submission and believe that we have an appropriate level of expertise to confirm that it is of an acceptable scientific standard, however we have significant reservations, as outlined above.

# Reviewer report: Reviewer response for version 1

**DOI:** 10.5256/f1000research.163172.r322293

**Published:** 2024-10-08

**Authors:** Sabar Sabar

**Affiliations:** 1 Sepuluh Nopember Institute of Technology, Surabaya, Indonesia

H1. 

 Explain why and how motivation (both intrinsic and extrinsic) specifically can affect absorptive capability. The relationship that needs to be established is Motivation to absorptive capability, while learning alone does not describe capability. This will provide a stronger basis for the hypothesis.

 H2.

 1. Explain further how the key components of transformational leadership (e.g., idealized influence, inspirational motivation, intellectual stimulation, and individualized consideration) can specifically enhance absorptive capability.

 2. Specifically explain how transformational leadership helps in the process of recognizing, assimilating, and applying new knowledge. This will help provide a stronger argument for why transformational leadership affects absorptive capability..

 Please use the suggestions for the next development hypothesis.

 Research Method

 In the research method, please add a common method bias referring to Podsakoff et al, 2013. (Ref 1)

 Discussion

 Research findings do not only refer to theoretical findings, but it is necessary to add how the findings are relevant in the context of the research being studied

Is the work clearly and accurately presented and does it cite the current literature?

Partly

If applicable, is the statistical analysis and its interpretation appropriate?

Yes

Are all the source data underlying the results available to ensure full reproducibility?

Yes

Is the study design appropriate and is the work technically sound?

Yes

Are the conclusions drawn adequately supported by the results?

Yes

Are sufficient details of methods and analysis provided to allow replication by others?

Yes

Reviewer Expertise:

Human Resources Strategy and Change Management

I confirm that I have read this submission and believe that I have an appropriate level of expertise to confirm that it is of an acceptable scientific standard, however I have significant reservations, as outlined above.

## References

[1] : Common method biases in behavioral research: a critical review of the literature and recommended remedies. *J Appl Psychol* .2003;88(5) : 10.1037/0021-9010.88.5.879 879-903 10.1037/0021-9010.88.5.879 14516251

# Response

**Published:** 2024-10-10

**Authors:** Eduard Sinaga

**Affiliations:** PSDM, Universitas Airlangga, Surabaya, East Java, Indonesia

H1. 

 Explain why and how motivation (both intrinsic and extrinsic) specifically can affect absorptive capability. The relationship that needs to be established is Motivation to absorptive capability, while learning alone does not describe capability. This will provide a stronger basis for the hypothesis.

 Answer:

 Through a number of pathways, motivation—both intrinsic and extrinsic—has a major impact on absorptive ability. People that are intrinsically motivated are naturally curious to learn new things, which prompts them to interact with new material more deeply. Goal-oriented behavior can be stimulated by extrinsic motivation, which also promotes active application and information acquisition. These motivational elements improve both behavioral and cognitive characteristics, including proactive information seeking and experimentation, as well as cognitive functions like attention allocation and elaborative processing. Crucially, motivation affects a person's ability to identify important new information, integrate it with prior knowledge, and use it effectively in their workplace. This goes beyond simple learning. The hypothesis that links motivation to absorptive capacity is strengthened theoretically by this all-encompassing influence on the fundamental elements of absorptive capacity (acquisition, assimilation, transformation, and exploitation of knowledge). This emphasizes that motivation impacts not only learning but the entire process of knowledge absorption and utilization

 H2.

 1. Explain further how the key components of transformational leadership (e.g., idealized influence, inspirational motivation, intellectual stimulation, and individualized consideration) can specifically enhance absorptive capability.

 2. Specifically explain how transformational leadership helps in the process of recognizing, assimilating, and applying new knowledge. This will help provide a stronger argument for why transformational leadership affects absorptive capability..

 Answer 1

 Motivation affects absorptive ability through promoting thought patterns and actions that are essential for acquiring and applying knowledge. Extrinsic motivation promotes proactive knowledge seeking and application, but intrinsic motivation fosters curiosity and deeper engagement with new information. These elements have a direct impact on a person's capability to identify, absorb, process, and apply knowledge—the fundamental elements of absorptive capacity. This affects not just learning but the entire process of assimilating knowledge and applying it effectively in professional settings. This strengthens the case for the hypothesis that motivation and absorptive capacity are positively correlated.

 Answer 2

 Through its essential elements, transformational leadership improves absorptive capacity: idealized influence stimulates knowledge seeking, inspirational motivation harmonizes learning with objectives, intellectual stimulation boosts knowledge transformation, and tailored consideration facilitates knowledge exploitation. This type of leadership makes it easier to identify new information by promoting environmental scanning, aids in knowledge assimilation through candid communication, and encourages experimentation to enhance knowledge application. Transformational leadership immediately affects the entire process of absorptive capacity by forming organizational culture and structures that support knowledge absorption. This makes a compelling case for transformational leadership's beneficial impact on absorptive capability.

 Question: Please use the suggestions for the next development hypothesis.

 Answer:

  The specific mechanisms by which motivation and transformational leadership improve absorptive capacity are now included in these created hypotheses, giving your research a stronger theoretical foundation.

 Question:

 Research Method

 In the research method, please add a common method bias referring to Podsakoff et al, 2013. (Ref 1)

 Answer:

 We took the advice of Podsakoff et al. (2013) to address any common technique bias. Procedures like time separation of data gathering and anonymity guarantee were put into place. In our structural equation model, we incorporated a common latent factor and performed statistical analyses using Harman's single-factor test. The marker variable technique was also utilized by us. These steps are intended to reduce typical procedure bias and improve the reliability of our results.

 Discussion

 Question

 Research findings do not only refer to theoretical findings, but it is necessary to add how the findings are relevant in the context of the research being studied

 Answer:

 We place our results in the perspective of the independent oil and gas sector in the Discussion. The significant moderating effect of absorptive capacity highlights how crucial it is for independent contractors to acquire and apply information in order to adapt to a variety of project types. Through absorptive capacity, the indirect effects of leadership and motivation on inventive behavior reveal intricate routes for innovation in this industry. These observations point to doable tactics that businesses may implement to boost freelancer creativity, like encouraging cultures of information sharing and offering a variety of learning opportunities. Our theoretical contributions gain depth from this industry-specific interpretation, which also provides practitioners in the sector with practical insights

# Reviewer report: Reviewer response for version 1

**DOI:** 10.5256/f1000research.163172.r294191

**Published:** 2024-07-05

**Authors:** Udin Udin

**Affiliations:** 1 Department of Managment, Universitas Muhammadiyah Yogyakarta, Yogyakarta, Special Region of Yogyakarta, Indonesia

This paper need major revision:

 INTRODUCTION

 1. It should clearly articulate the gap in the existing literature that the study aims to address. Providing a more explicit statement of the research problem or gap would help readers understand the significance of the study.

 2. It would be beneficial to highlight the specific novelty or contribution of the study to the field of absorptive capability, motivation, and leadership research, especially in the context of freelancers in the oil and gas industry.

 METHODOLOGY

 1. Providing more detailed information on the methodology used to assess validity and reliability, including specific statistical tests and results, would enhance the transparency and reproducibility of the study.

 2. While the study mentions the number of participants (250 freelancers), additional information on the sample size justification and power analysis would strengthen the validity of the study results.  

 3. The study reports various model fit indices such as RMSEA, NFI, RFI, IFI, TLI, CFI, RMR, GFI, AGFI, and PGFI. While these indices provide insights into the model fit, it would be beneficial to provide a detailed explanation of each index, its interpretation, and the thresholds used to determine model fit.

 DISCUSSION

 1. It should provide a thorough interpretation of the study findings in relation to the research questions or objectives. Clearly explaining how the results align with the study's hypotheses or expectations would enhance the clarity of the discussion.

 2. It is important to avoid simply restating the results but instead focus on explaining the significance of the findings in the context of absorptive capability, motivation, and leadership in freelancers in the oil and gas industry.

 3. Addressing any discrepancies or unexpected results by comparing them with established theories or empirical evidence can offer valuable insights into the complexity of the relationships between absorptive capability, job motivation, transformational leadership, and innovation work behavior. 

 These recent readings are strongly recommended:

 a) (Udin, 2024)(Ref-1)

 b) (Udin et al., 2024)(Ref-2)

 c) (Udin et al.,2022))(Ref-3)

 CONCLUSION

 1. It should clearly outline the practical implications of the study findings for organizations, leaders, and freelancers in the oil and gas industry. Providing actionable recommendations based on the results can enhance the relevance of the research.

 2. Discussing how the identified relationships between absorptive capability, job motivation, and transformational leadership can be leveraged to improve innovative work behavior and organizational performance would add depth to the practical implications.

Is the work clearly and accurately presented and does it cite the current literature?

Yes

If applicable, is the statistical analysis and its interpretation appropriate?

Partly

Are all the source data underlying the results available to ensure full reproducibility?

Yes

Is the study design appropriate and is the work technically sound?

Yes

Are the conclusions drawn adequately supported by the results?

Partly

Are sufficient details of methods and analysis provided to allow replication by others?

Yes

Reviewer Expertise:

Leadership Studies; Islamic Business Ethics; Knowledge Management; Innovative Work Behavior; Organizational Effectiveness

I confirm that I have read this submission and believe that I have an appropriate level of expertise to confirm that it is of an acceptable scientific standard.

## References

[1] : Leadership styles and innovative work behaviour: the role of work engagement. *https://www.inderscienceonline.com/doi/abs/10.1504/IJEBR.2024.139287* .2024;

[2] : Exploring three-mediation paths of transformational leadership and innovative work behavior. *https://archive.conscientiabeam.com/index.php/35/article/view/3615* .2024;

[3] : Transformational Leadership and Innovative Work Behavior: Testing the Mediating Role of Knowledge Sharing and Work Passion. *https://journal.unnes.ac.id/nju/jdm/article/view/34446* .2022;
